# Rag1^−/−^ Mutant Zebrafish Demonstrate Specific Protection following Bacterial Re-Exposure

**DOI:** 10.1371/journal.pone.0044451

**Published:** 2012-09-06

**Authors:** Claudia Hohn, Lora Petrie-Hanson

**Affiliations:** Department of Basic Sciences, College of Veterinary Medicine, Mississippi State University, Mississippi State, Starkville, Mississippi, United States of America; INRA, France

## Abstract

**Background:**

Recombination activation gene 1 deficient (rag1^−/−^) mutant zebrafish have a reduced lymphocyte-like cell population that lacks functional B and T lymphocytes of the acquired immune system, but includes Natural Killer (NK)-like cells and Non-specific cytotoxic cells (NCC) of the innate immune system. The innate immune system is thought to lack the adaptive characteristics of an acquired immune system that provide enhanced protection to a second exposure of the same pathogen. It has been shown that NK cells have the ability to mediate adaptive immunity to chemical haptens and cytomegalovirus in murine models. In this study we evaluated the ability of rag1^−/−^ mutant zebrafish to mount a protective response to the facultative intracellular fish bacterium *Edwardsiella ictaluri*.

**Methodology/Principal Findings:**

Following secondary challenge with a lethal dose of homologous bacteria 4 and 8 weeks after a primary vaccination, rag1^−/−^ mutant zebrafish demonstrated protective immunity. Heterologous bacterial exposures did not provide protection. Adoptive leukocyte transfers from previously exposed mutants conferred protective immunity to naïve mutants when exposed to homologous bacteria.

**Conclusions/Significance:**

Our findings show that a component of the innate immune system mounted a response that provided significantly increased survival when rag1^−/−^ mutant zebrafish were re-exposed to the same bacteria. Further, adoptive cell transfers demonstrated that kidney interstitial leukocytes from previously exposed rag1^−/−^ mutant zebrafish transferred this protective immunity. This is the first report of any rag1^−/−^ mutant vertebrate mounting a protective secondary immune response to a bacterial pathogen, and demonstrates that a type of zebrafish innate immune cell can mediate adaptive immunity in the absence of T and B cells.

## Introduction

The immune system of fish provides important information about conserved processes in the mammalian immune system. Studies of antibody production in fish have revealed much about the phylogeny of acquired immunity and immunoglobulins and this has led to a better understanding of the overall functionality of the immune system in all vertebrates (reviewed in [1], [2]). Fish are an excellent model for studying innate immunity since their innate immune components are homologous to those of mammals. Their acquired immunity differs from more advanced vertebrates in the length of time needed to initiate a specific immune response because of their poikilothermic nature [3]. Furthermore, other than the mouse model, the rag1^−/−^ mutant zebrafish is the only animal model available for investigating T and B cell deficient immunological responses. Interestingly, fish are not immunologically mature when they hatch. Acquired immunity, utilizing fully functional T and B cells, does not develop until 3 to 6 weeks post-hatch, depending on the species. In previous work channel catfish (*Ictalurus punctatus*) larvae that had minimally organized lymphoid tissue produced a protective secondary response to a bacterial pathogen [4], [5], [6]. This suggested that in fish, there is an adaptive component to innate immunity.

Historically, immunological dogma described the innate immune response as acting naïvely to each encounter with a pathogen depending on the recognition of conserved molecular patterns and exhibiting only weak specificity. T and B cells mediate protective secondary immune responses of the acquired immune system, and immune-deficiencies develop in their absence. However, evidence of adaptive responses of cells of the innate immune system of mice to haptens and cytomegalovirus have been demonstrated [7], [8]. Natural Killer cells, an innate lymphocyte population, can mount antigen-specific immunological memory [9], [10], [11], [12], [13]. Innate immune system memory may be present in, and a more critical component of, lower vertebrate immunity.

We used zebrafish (*Danio rerio*) to investigate an adaptive component of innate immunity because specific mutants are available and zebrafish are recognized as infectious disease models [14], [15], [16]. Numerous regions of synteny between the zebrafish and human genomes have been identified [17] allowing immunological findings in zebrafish to be translated to higher vertebrates. Rag1**^−/−^** mutant zebrafish created by a reverse genetic approach have been shown to lack VDJ recombination [18]. After establishing a breeding colony, we further characterized the rag1^−/−^ mutant zebrafish to confirm lack of T cell receptor (TCR) and immunoglobulin (Ig) transcript expression [19]. Therefore these fish do not have mature T and B cells and thus are a unique model for characterizing innate immune system memory in fish.

In this study, rag1^−/−^ mutant zebrafish were given a primary (vaccination) exposure to a low dose of a bacterial pathogen, or a sham exposure. To determine protection, or how well the vaccination worked, a secondary high dose exposure was delivered at either four weeks (Trial 1) or eight weeks (Trial 2) after the primary. The pathogens used were members of the Enterobacteriaceae family: *Edwardsiella ictaluri, Yersinia ruckeri* and *E. tarda*. *Edwardsiella ictaluri* causes Enteric Septicemia of Catfish and a comparable disease in zebrafish [14]. *Edwardsiella ictaluri* RE-33 is an attenuated live vaccine **(**AQUAVAC-ESC® Intervet, Inc.**)** [20] that was used for the vaccination (primary) exposure of *E. ictaluri*. *Yersinia ruckeri* causes Yersiniosis or Enteric Red Mouth Disease (ERM) primarily in salmonids [21]. Infection trials in our lab demonstrated susceptibility of zebrafish to *Y. ruckeri* following intramuscular (IM) injection. *Edwardsiella tarda* produces localized and systemic infections in a wide variety of vertebrates and has been shown to establish infections in zebrafish [22]. The aim of this study was to utilize T and B cell deficient rag1^−/−^ mutant zebrafish to investigate adaptive protection of the innate immune system in response to repeated bacterial exposure.

**Table 1:** Summary of the trials performed to determine the basis of protection following bacterial re-exposure in rag1^−/−^ mutant zebrafish.

Trial	Primary	Treatment; Objective; # of experiments; tanks/treatment; fish/tank	Secondary
1	*E. ictaluri* RE33 10^4^	4 week interval; Determine if protection occurs; 3 exps; 3 tanks/trt; 24 fish/tank	*E. ictaluri* 10^4^
2	*E. ictaluri* RE33 10^4^	8 week interval; Rule out temporarily heightened primary effects; 2 exps; 8 tanks/trt;10 fish/tank	*E. ictaluri* 10^4^
3	*E. ictaluri* RE33 10^4^	4 week interval with antibiotic administered; Rule out persistent pathogen presence;2 exps; 8 tanks/trt; 10 fish/tank	*E. ictaluri* 10^4^
4	*E. ictaluri* RE33 10^4^	4 week interval with homologous or heterologous secondary; Determine specificity;1 exp; 4 tanks/trt; 15 fish/tank	*E. ictaluri* 10^4^ *Y. ruckeri* 10^6^ *E. tarda* 10^2^
5	*E. ictaluri* RE33 10^4^	4 week interval with adoptive cell transfers; determine if protection provided bya leukocyte population; 1 exp; 7 tanks/trt; 15 fish/tank	*E. ictaluri* 10^4^

## Materials and Methods

### Animal Care

Rag1^−/−^ mutant zebrafish were housed in the Mississippi State University College of Veterinary Medicine (MSU-CVM) specific pathogen free (SPF) fish hatchery. Fish were propagated according to modified standard protocols posted at: http://www.cvm.msstate.edu/zebrafish/index.html. The Institutional Animal Care and Use Committee at Mississippi State University approved all experimental animal protocols.

### Genotype Control

All rag1^−/−^ mutant zebrafish used for this study were bred at the CVM-SPF fish hatchery. We established a homozygous rag1^−/−^ mutant zebrafish breeding colony, and all of our experimental fish are progeny from this colony. For an additional genotype control we sub-sampled zebrafish (10 fish per spawn) shortly before the experiment and the genotype, rag1^−/−^, was confirmed using previously established PCR protocols [19].

**Figure 1:**
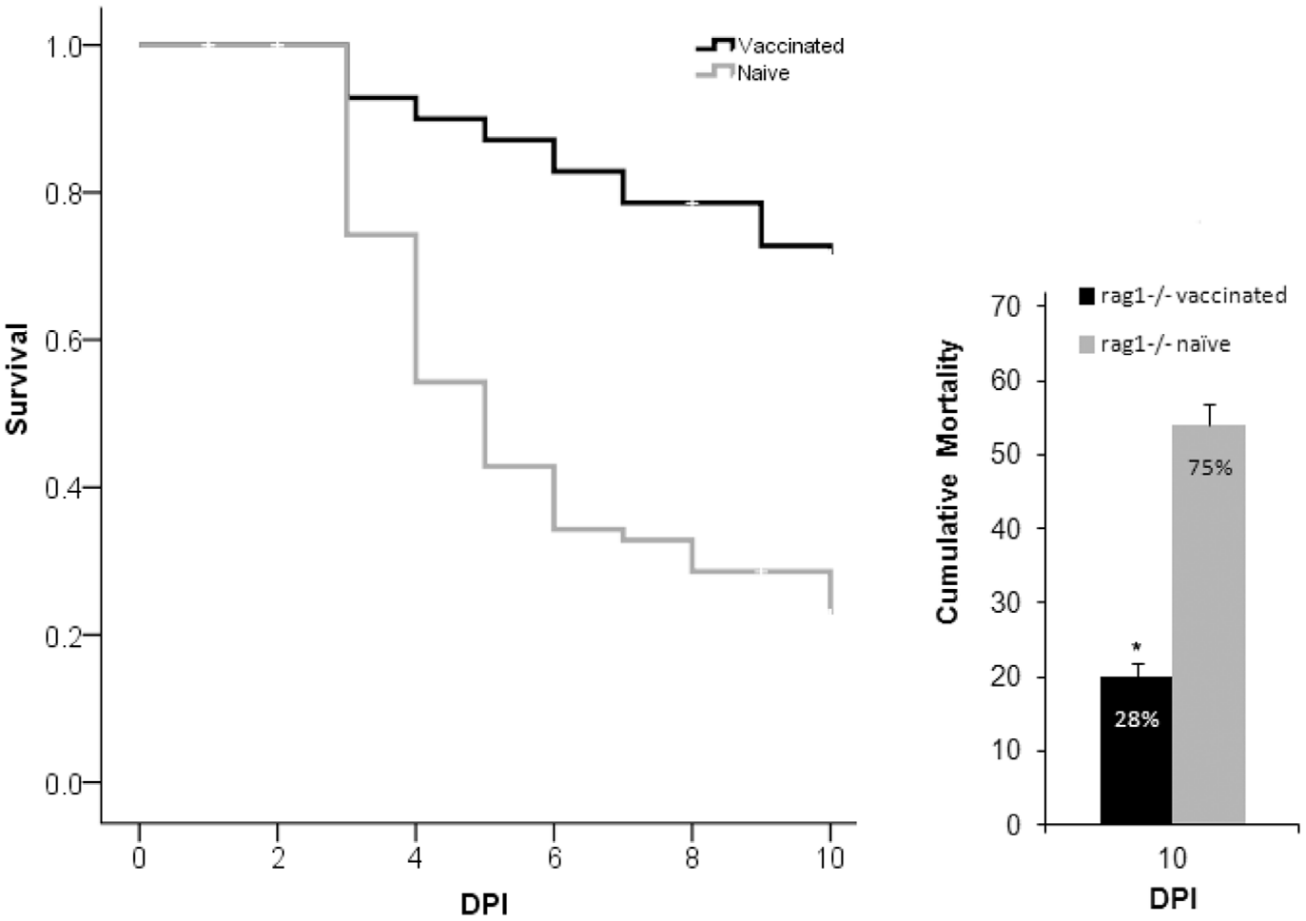
Comparison of survival by day (left panel) and cumulative mortality (right panel) between naïve and vaccinated rag1^−/−^ mutant zebrafish in Trial 1. Asterisk indicates a significantly lower mortality rate in vaccinated rag1^−/−^ mutant zebrafish when compared to naïve rag1^−/−^ mutant zebrafish. Fish were vaccinated with RE33® an attenuated strain of *E. ictaluri* and 4 weeks later challenged by a secondary injection of virulent *E. ictaluri*. DPI = days post secondary exposure. Error bars indicate standard error of the mean, SEM, between tanks (n = 9).

### Preparation of Bacterial Cultures

*Edwardsiella ictaluri*, *Yersinia ruckeri* and *Edwardsiella tarda* were case isolates from fish submitted to the Fish Diagnostic Lab at CVM-MSU. Culture identifications were confirmed by biochemical analysis using the bioMerieux api20E strip (BioMerieux, 69280 Marcy l’Etoile, France). Aliquots (0.5 ml) were stored in 20% glycerol at −80°C until needed for trials, at which time one aliquot was thawed and added into Brain Heart Infusion broth and incubated in a shaker incubator at 30°C overnight. Logarithmic phase cultures were obtained by dilution of the overnight culture 1∶10 and grown until the optical density was 0.4 at 540 nm which corresponds to 10^8^ colony forming units (CFU) per ml. Culture purities were assessed and bacterial concentrations determined by plating serial dilutions on 5% sheep blood plates.

### Lethal Dose (LD>80) Determination

In separate trials, rag1^−/−^ mutant zebrafish were injected with *E. ictaluri*, *Y. ruckeri,* or *E. tarda* (10^6^, 10^5^, 10^4^, 10^3^, 10^2^, or 10^1^ CFU/fish) to determine LD_>80_ dosage for the secondary exposure, referred to as the protection exposure (to determine if the vaccination exposure provided protection). Injections of rag1^−/−^ mutant zebrafish were performed using four replicate tanks per treatment with 15 fish per replicate. Additionally 15 control fish per strain were sham injected. Mortalities were recorded for 18 days post injection (dpi), and LD_>80_ dosages were determined from these data (Figure S1).

**Figure 2:**
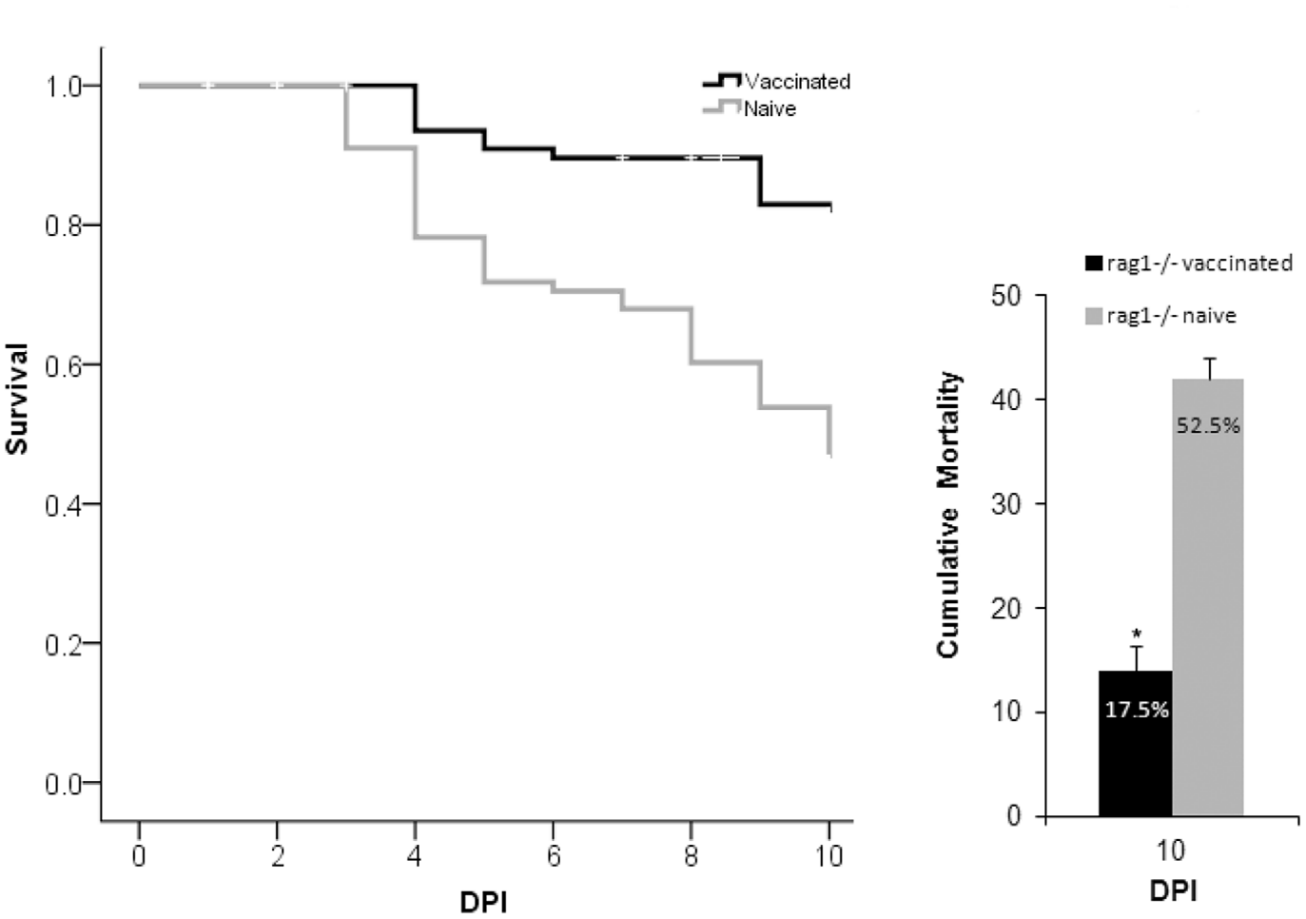
Comparison of survival by day (left panel) and cumulative mortality (right panel) between naïve and vaccinated rag1^−/−^ mutant zebrafish in Trial 2. Asterisk indicates a significantly lower mortality rate in vaccinated rag1^−/−^ mutant zebrafish when compared to naïve rag1^−/−^ mutant zebrafish. Fish were vaccinated with RE33® an attenuated strain of *E. ictaluri* and 8 weeks later challenged by a secondary injection of virulent *E. ictaluri*. DPI = days post secondary exposure. Error bars indicate standard error of the mean, SEM, between tanks (n = 16).

### Bacterial Injections and Experimental Observations

Primary and secondary injections were carried out as described in Table 1. In all trials, adult (6–9 month old) rag1^−/−^ mutant zebrafish were anesthetized in 110 mg/L buffered tricaine methane sulfonate (MS222). Each fish was IM injected on the lateral line above the anal fin using an insulin syringe. All primary vaccinations were 10^4^ CFU/fish RE33, a commercial, live, attenuated *Edwardsiella ictaluri* vaccine strain (AQUAVAC-ESC® Intervet, Inc.) [20]. The secondary exposure was delivered at either 1 month (Trial 1) or 2 months (Trial 2) post-vaccination and consisted of one of the following bacteria: 10^4^ CFU/fish *Edwardsiella ictaluri*, 10^6^ CFU/fish *Yersinia ruckeri*, or 10^2^ CFU/fish *Edwardsiella tarda* (Table 1). These dosages have been established in our laboratory as the LD_>80_ dosage for each bacteria. This secondary injection is referred to as protection exposure (to determine if the vaccination exposure provided protection). All injections were delivered in a total volume of 10 µl phosphate buffered saline (PBS). Sham injected fish received 10 µl PBS. After recovery from anesthesia, fish were moved to tanks in a flow–through water system and maintained at 27°C±1°. All fish were held under the same conditions during all experiments and were observed 3 times a day for clinical signs of disease. Moribund fish were euthanatized in 340 mg/L MS222, and sampled for bacterial re-isolation. Mortalities were recorded for 10 days post-protection exposure.

### Re-isolation of Bacteria

After protection exposures, deaths were recorded, and a 10 µl loop of each dead fish’s brain was plated on 5% sheep blood plates. After 24 to 48 h at 28°C, bacterial identifications were confirmed by biochemical analysis using the bioMerieux api20E strip (BioMerieux, 69280 Marcy l’Etoile, France).

**Figure 3:**
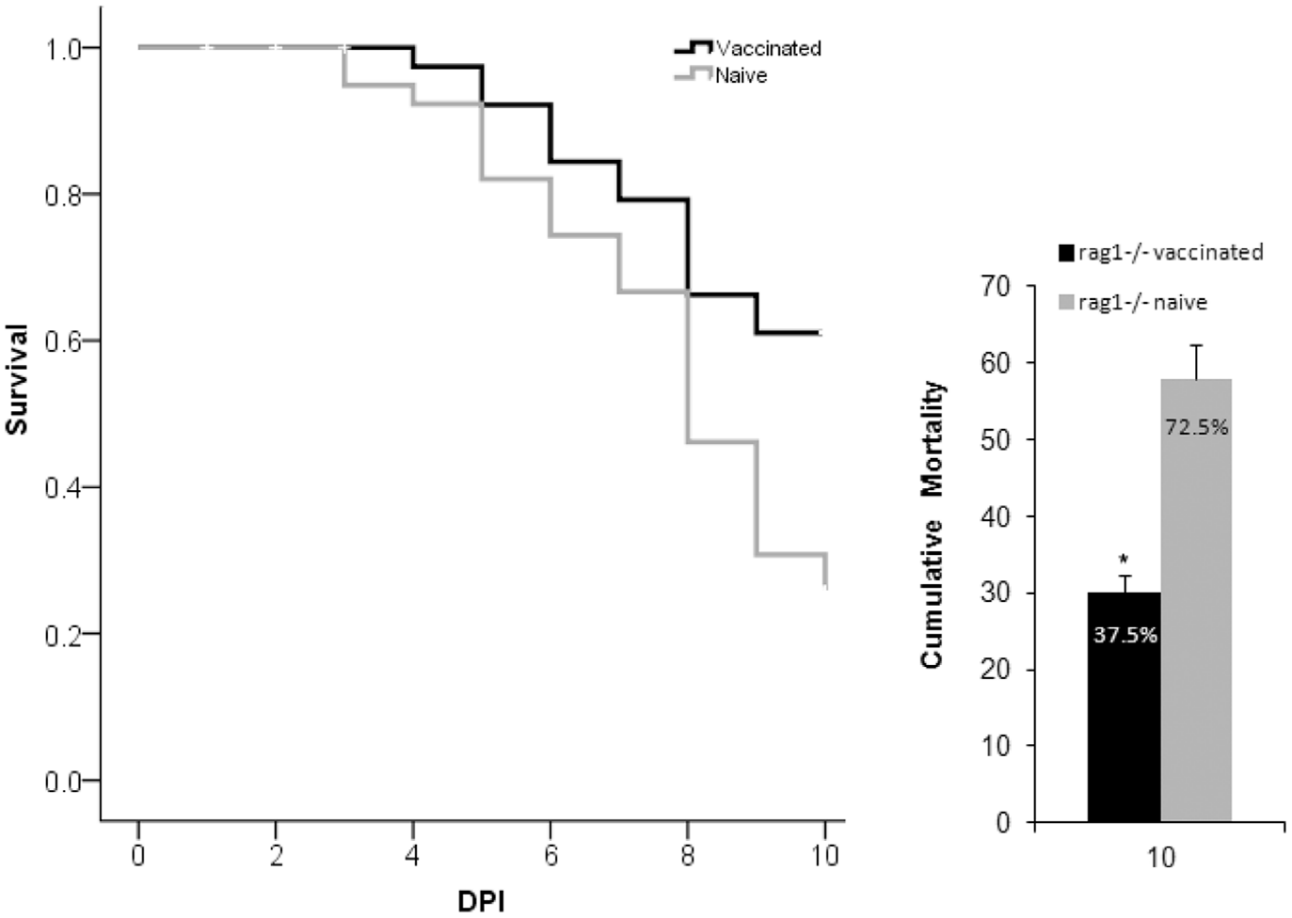
Comparison of survival by day (left panel) and cumulative mortality (right panel) between naïve and vaccinated rag1^−/−^ mutant zebrafish fed antibiotic feed in Trial 3. Asterisk indicates a significantly lower mortality rate in vaccinated rag1^−/−^ mutant zebrafish when compared to naïve rag1^−/−^ mutant zebrafish. Fish were vaccinated with RE33® an attenuated strain of *E. ictaluri* and 4 weeks later challenged by a secondary injection of virulent *E. ictaluri*. Ten days after the vaccination, fish received feed supplemented with oxolinic acid for 7 days. DPI = days post secondary exposure. Error bars indicate standard error of the mean, SEM, between tanks (n = 16).

### Overview of Exposure Trials

Five different trials were designed to progressively determine the basis of adaptive immunity in rag1^−/−^ mutant zebrafish (Table 1). Lethal Dose trials determined the dose of bacteria per fish required to kill 80% of a naïve population in the secondary exposure. Throughout Trials 1–4 we followed the general set-up as outlined in Table 1. For negative controls, fish were injected with sterile PBS (sham). For each trial, a tank of non-injected sentinel fish was also included. Since infected fish can shed bacteria, all tanks were on flow-through (0.5 L/min) throughout the experiments to ensure good water quality and to minimize the risk of bacterial accumulation. In a previous study we showed that immersion exposed zebrafish did not establish an infection when exposed to 10^5^ CFU/mL of tank water for 2 hours [14]. Therefore, the likelihood of bacterial transmission through the water is very low.

In Trial 1, we compared the susceptibility and adaptive protection of rag1^−/−^ mutant zebrafish when challenged with *E. ictaluri*. For the primary vaccination exposure, rag1^−/−^ mutant zebrafish were injected with 10^4^ CFU/fish of the attenuated strain of RE33 *E. ictaluri*, or PBS only (controls). For the secondary or protection exposure, rag1^−/−^ mutant zebrafish were injected with 10^4^ CFU of *E. ictaluri*/fish 4 weeks after the vaccination exposure. Naïve rag1^−/−^ mutant zebrafish were also injected with 10^4^ CFU/fish *E. ictaluri*. Negative controls included mutants injected with PBS only at the primary and secondary exposure, and mutants that were not injected at all.

**Figure 4:**
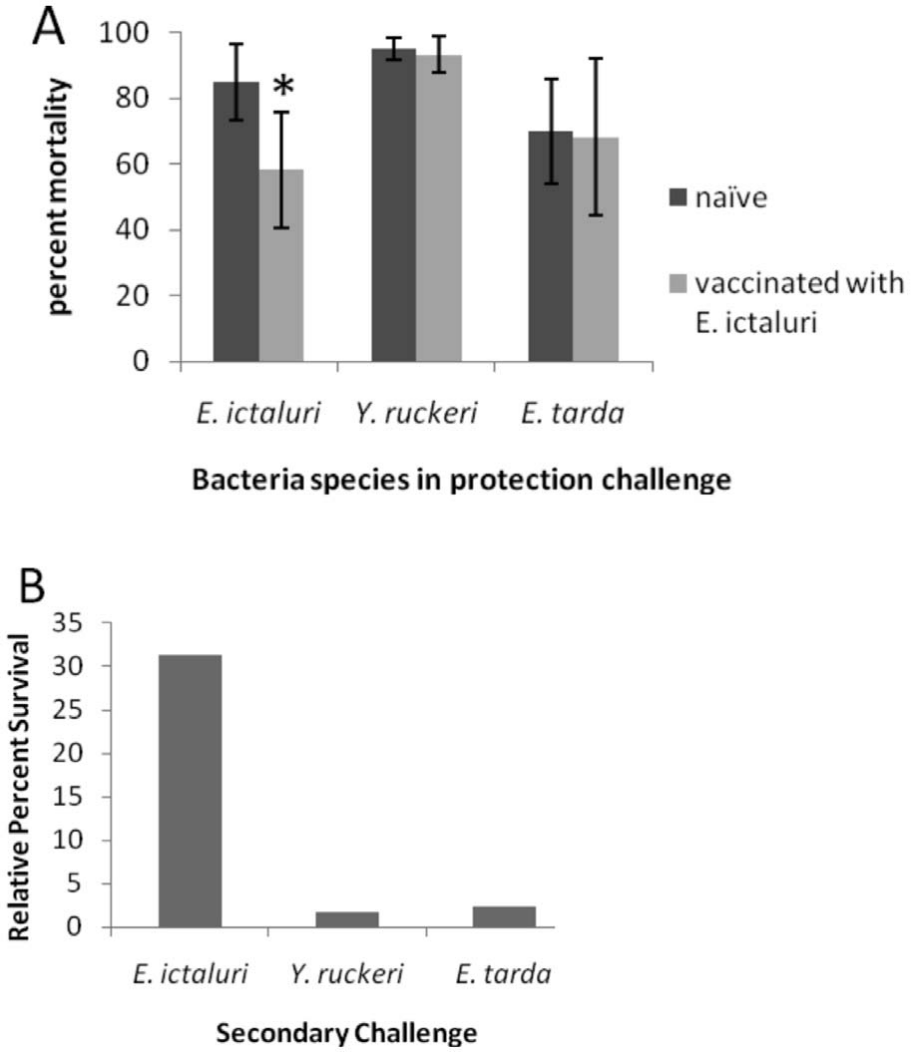
Homologous and heterologous protection trial in Trial 4. Percent survival, A, and relative percent survival [(1– mean mortality of vaccinated/mean mortality of sham vaccinated) × 100] B, of *E. ictaluri* RE33® vaccinated rag1^−/−^ mutant zebrafish after secondary challenge with *E*. *ictaluri*, *Y. ruckeri* or *E. tarda.* Error bars indicate standard deviation between tanks (n = 4).

It is known that for a short time after the primary exposure the innate immune response is heightened, and if that heightened response is present when the secondary exposure is given, protection could be non-specific. To rule out this effect in Trial 2, we performed the same experiment as in Trial 1, except we increased the time between the vaccination and protection exposures to eight weeks. Set-up and procedures performed were the same as described in Trial 1.

Trial 3 was performed to confirm that there was not a persistent low-level infection that was continually stimulating the innate immune system. In previous trials, sub-samples of vaccinated fish were cultured for bacteria and found to be negative, but a low level of infection may escape detection. Therefore, we administered oxolinic acid, an antibiotic used as a feed additive in fish. The fish received feed supplemented with oxolinic acid (7 mg/gm) daily for 7 days (fed to satiation) starting at 10 days post the primary vaccinations.

**Figure 5:**
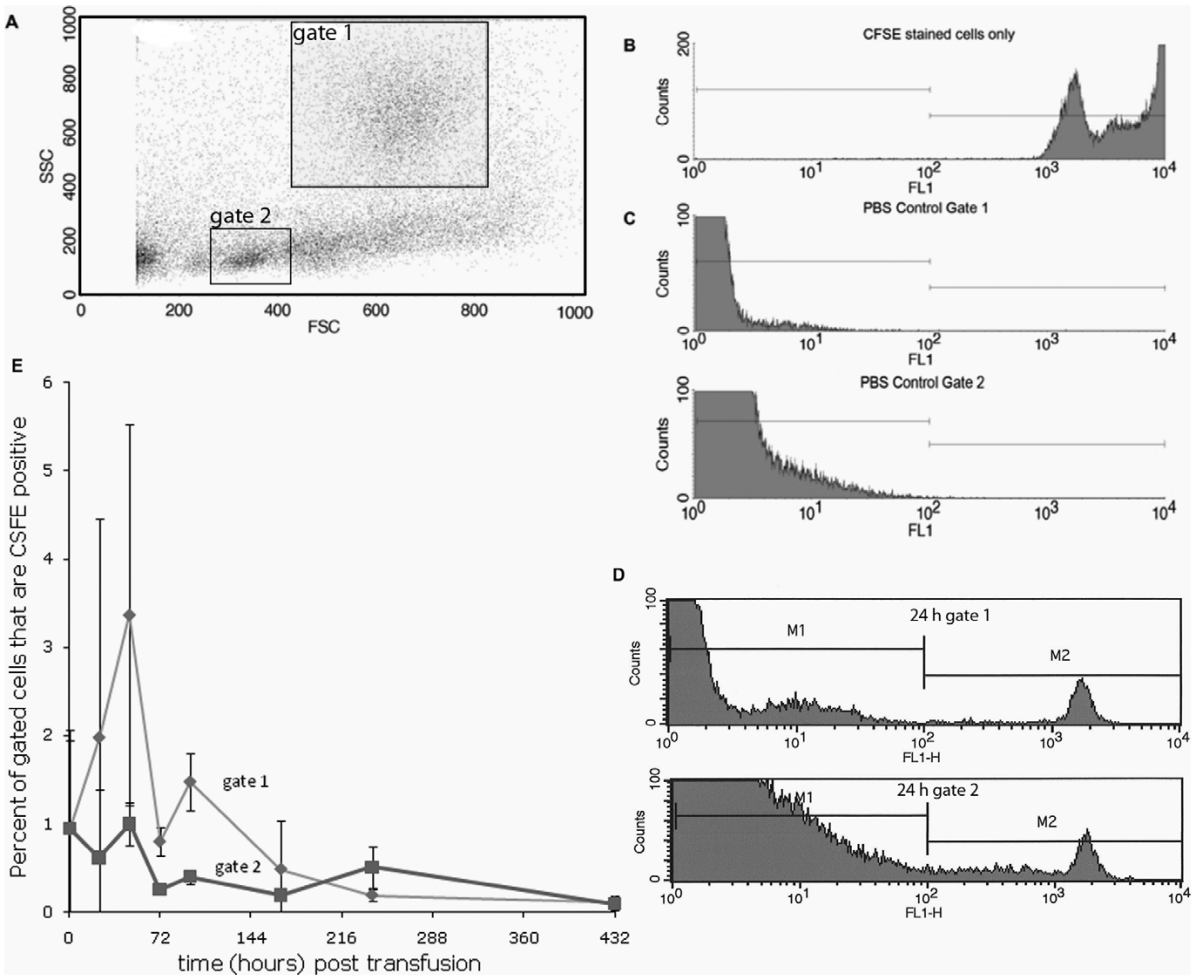
Flow cytometry data of CFSE stained leukocytes re-isolated from recipient rag1^−/−^ mutant kidneys. **A.** Scatter plot of kidney leukocytes demonstrating the gating of cell populations (gate 1 is phagocytes, gate 2 is lymphocyte like cells). **B.** Histogram of positive control: CFSE stained cells injected into the recipient. **C.** Histogram of negative control: cells isolated from a PBS injected fish. **D.** Histograms CSFE stained cell counts from gate 1 (phagocytes) and gate 2 (lymphocyte like) from kidney tissue of recipient rag1^−/−^ mutants at 24 h post infusion. **E.** Graph indicating the CFSE stained cell counts in the kidney of transfused fish at 1 h, then 1, 2, 3, 4, 7 and 18 days post transfusion. Error bars indicate standard deviation (n = 2).

In Trial 4, we determined the specificity of protection by performing heterologous (different bacteria species in primary and secondary exposures) bacteria challenges. Rag1^−/−^ mutant zebrafish were vaccinated with 10^4^ CFU *E. ictaluri* RE33/fish or sham injected (Table 1). Four weeks later these fish were injected with 10^4^ CFU *E. ictaluri*/fish, 10^6^ CFU *Y. ruckeri*/fish or 10^2^ CFU *E. tarda*/fish; these were dosages that resulted in greater than 80% mortality of naïve fish in Lethal Dose trials. Post-exposure procedures were the same as described in the other trials.

Trial 5 was performed to determine if we could transfer protection by transferring innate immune cells from vaccinated rag1^−/−^ mutant zebrafish into naïve rag1^−/−^ mutant zebrafish before they were exposed to bacteria. In addition to blood filtering functions, fish kidney tissue is functionally equivalent to vertebrate bone marrow and is a primary lymphoid tissue. It consists of renal corpuscles and collecting tubules with an interstitial matrix of hematopoietic tissue that includes hematopoietic stem cells, macrophages, neutrophils, NCC and NK cells in rag1*^−/−^* mutant zebrafish [19]. To validate adoptive cell transfer experiments in rag1^−/−^ mutant zebrafish, we performed preliminary transfer experiments using carboxyfluorescein diacetate succinimidyl ester (CSFE) labeled cells. The mutant zebrafish population used in this trial was in-bred for 5 generations to minimize recognition of transfused cells by the recipient.

**Figure 6:**
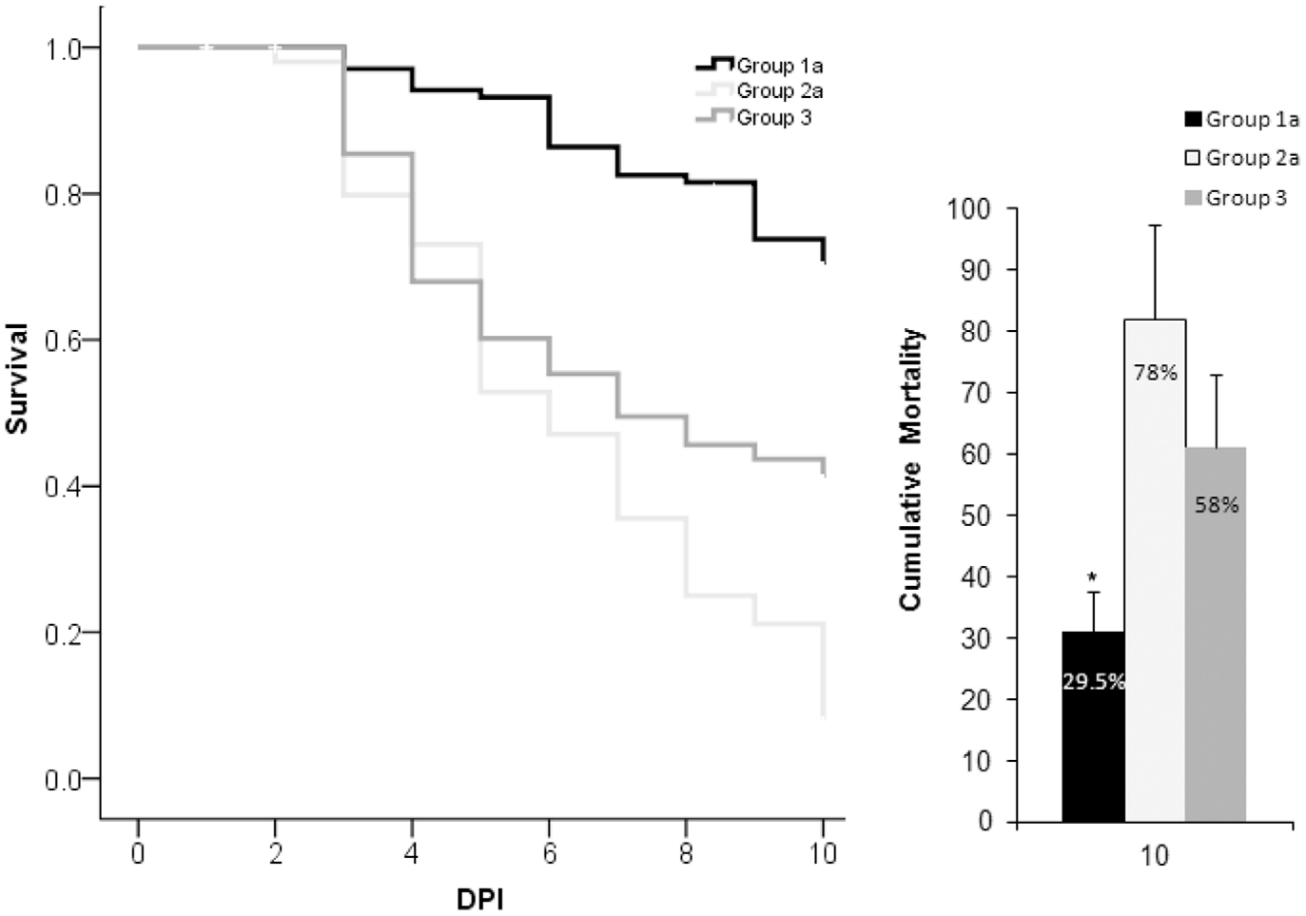
Comparison of survival by day (left panel) and cumulative mortality (right panel) in Adoptive Immunity Trial 5. Asterisk indicates that recipients of vaccinated transferred kidney interstitial cells (Group 1a) had significantly lower (p<0.05) mortality than recipients of naïve transferred cells (Group 2a), or naïve fish injected with *E. ictaluri* (Group 3). DPI = days post injection of the secondary exposure. Error bars indicate standard deviation between tanks (n = 7).

### Preparation and Recovery of Carboxyfluorescein Diacetate Succinimidyl Ester (CFSE) Stained Donor Cell Suspensions

Kidney tissue was dissected from 10 rag1^−/−^ mutant zebrafish and prepared following established procedures in our lab [23]. The cell suspension was diluted to 10^6^ cells/ml in PBS. The CFSE probe was prepared according to manufacturer’s directions (CellTrace™ CFSE Cell Proliferation Kit (C34554) Molecular Probes™). Five mM of CFSE probe per ml of cell suspension was added, and then incubated for 15 min at 30°C. The cell suspension was centrifuged at 400 g for 5 min, and the cell pellet re-suspended in tissue culture medium to obtain 10^8^ cells/ml. CFSE stained cells (10 µl) were injected intraperitoneally (IP) into recipient rag1^−/−^ mutant zebrafish delivering 10^6^ cells/fish. Control fish were injected with 10 µl of PBS. Fish were held in flow through tanks until sampled, and recipient kidneys were sampled as previously described. Flow cytometric analyses of adopted cells were performed on 3 replicates of adopted stained cells and 3 control replicates at 1, 24, and 48 hours, and 3, 4, 7, 10, 18 days post-injection.

In Trial 5, we performed adoptive transfers of renal interstitial cells from vaccinated and naïve rag1^−/−^ mutant zebrafish (Figure S2). All adoptive transfers were carried out by infusing all the interstitial kidney leukocytes from the kidney of a single donor into a naïve recipient. Primary vaccinations were carried out as described in Trial 1 to provide donor rag1^−/−^ mutant zebrafish. Four weeks post-vaccination, kidneys of vaccinated rag1^−/−^ mutant zebrafish were removed, dissociated and cells were rinsed. Vaccinated donor leukocytes were IP injected into naïve recipient rag1^−/−^ mutant zebrafish (Group 1). Cells from naïve rag1^−/−^ mutant zebrafish were transferred into Group 2, and naïve control rag1^−/−^ mutant zebrafish did not receive cells (Group 3). Appropriate control groups included a transfused cell control group to rule out the effects of enhanced anti-bacterial activity resulting from non-specific cellular activation due to MHC or other transplantation antigen differences. Twenty-four hours later, fish received a secondary homologous exposure and were designated Groups 1a, 2a and 3. Control groups received PBS and were designated Groups 1b and 2b (Figure S2).

### Statistical Methods

Cumulative mortality was calculated in trials 1, 2, 3 and 5. Relative percent survival (RPS) [(1- mean mortality of vaccinated/mean mortality of sham vaccinated) x 100] was calculated for Trial 4, homologous and heterologous exposures, because RPS is a more robust analysis for protection to determine specificity. Treatment groups for each trial are described above. Number of tanks per treatment and number of fish per tank are shown in Table 1. Each tank was treated as a biological replicate. Mortality data of treatments between groups were analyzed by one-way analysis of variance (ANOVA) with post hoc Tukey HSD correction for multiple comparisons with a level of significance at p≤0.05. Statistical analyses were performed using SPSS for Windows 15.0 (SPSS Inc., Chicago, IL).

## Results

In Trial 1, a significantly lower cumulative mortality of 28% was seen in vaccinated rag1^−/−^ mutant zebrafish, while naïve rag1^−/−^ mutant zebrafish suffered a cumulative mortality of 75% (Figure 1). No losses occurred in the control fish and randomly selected rag1^−/−^ mutant zebrafish cultured negative for *E. ictaluri*. These findings demonstrate that in vaccinated rag1^−/−^ mutant zebrafish, a form of adaptive protection occurred.

In Trial 2, we saw similar results as in Trial 1. Cumulative mortality in naïve rag1^−/−^ mutant zebrafish was 52.5%. As expected, mortality of vaccinated rag1^−/−^ mutant zebrafish decreased significantly to 17.5% (Figure 2). These results demonstrate that protection in rag1^−/−^ mutant zebrafish is not due to a temporarily heightened post-primary response. The results also demonstrate that a component of innate immunity in rag1^−/−^ mutant zebrafish is capable of mediating adaptive protective immunity following vaccination.

In Trial 3, randomly sampled fish cultured negative for *E. ictaluri* before evaluating protection. This trial resulted in a similar significant cumulative mortality as in Trial 1 and 2. Vaccinated rag1^−/−^ mutant zebrafish demonstrated significantly greater protection with a cumulative mortality of 37.5% compared to 72.5% in naïve rag1^−/−^ mutant zebrafish (Figure 3). *Edwardsiella ictaluri* isolated from moribund fish post the secondary exposure were sensitive to oxolinic acid. These findings demonstrate that protection in the rag1^−/−^ mutant zebrafish was not due to bacteria persisting within the fish.

In Trial 4, rag1^−/−^ mutant zebrafish vaccinated with *E. ictaluri* demonstrated significantly reduced mortality following secondary *E. ictaluri* exposure, but were not protected against secondary *Y. ruckeri* or *E. tarda* exposures (Figure 4). Thus, homologous exposures provided specific adaptive protection whereas heterologous exposures did not.

To validate adoptive cell transfer experiments in rag1^−/−^ mutant zebrafish, we performed preliminary transfer experiments using carboxyfluorescein diacetate succinimidyl ester (CSFE) labeled cells. We modified standard procedures to perform adoptive cell infusions, or transfusions, in zebrafish. We successfully isolated kidney leukocytes, CFSE stained these cells, and IP injected them into recipient fish. After re-isolating stained cells from recipient kidney tissue and performing flow cytometric analyses on them, we compared their tissue distribution to naïve adopted cells. Our findings demonstrate that transfused donor cells were stable for greater than 7 days in recipient fish (Figure 5).

In Trail 5, group 1a received vaccinated cells and the cumulative mortality was significantly less (29.5%) when compared to rag1^−/−^ mutant zebrafish that received naïve cells, or no cells at all (Figure 6). Vaccinated kidney interstitial cells from rag1^−/−^ mutant zebrafish mediated specific adaptive protection in naïve recipients. Significantly higher cumulative mortalities were seen in exposed rag1^−/−^ mutant zebrafish that received naïve cells and exposed rag1^−/−^ mutant zebrafish that did not receive any cells, 78% and 58% respectively. Since these two treatments were not significantly different from each other, an increased number of naïve cells alone did not provide protection.

## Discussion

We have utilized T and B cell deficient rag1^−/−^ mutant zebrafish to investigate adaptive protection in response to bacterial infections in the absence of an acquired immune system. These mutant fish have increased numbers of neutrophils [19] and increased expression of genes associated with innate defenses [24]. This is the likely explanation for the ability of lymphocyte deficient zebrafish to resolve primary infection. More intriguing is our finding of significant differences in mortality upon secondary exposure, which demonstrates that rag1^−/−^ mutant zebrafish are able to develop and maintain protective immunity following a primary vaccination exposure. Furthermore, specificity was demonstrated when infection success was significantly reduced after previous contact with the same pathogen (homologous exposure), but not to a different pathogen (heterologous exposure). The cells that mediated specific recognition and a protective response upon secondary exposure performed these same functions when transferred into naïve rag1^−/−^ mutant zebrafish. Our finding of adaptive protection mediated by the innate immune system of zebrafish parallels similar findings in lymphocyte deficient mice. Furthermore, our findings are unique because they are the first demonstration of a T and B cell deficient rag1^−/−^ mutant vertebrate to mount an adaptive protective response to bacteria.

Natural Killer cells are the most likely innate immune cell mediating protective immunity in rag1^−/−^ mutant zebrafish. Natural Killer cell genes that encode pathogen recognition receptors do not undergo gene rearrangement [25]. Research on the functions of NK cells in mice have demonstrated adaptive, acquired immune responses [7]. A hapten-based hypersensitivity study was the first to suggest NK cells had the capacity of memory in *Rag*-2 deficient Severe Combined Immunodeficient (SCID) mice [7]. Severe Combined Immunodeficient mice possess NK cells but are devoid of T and B lymphocytes. These mice demonstrated substantial contact hypersensitivity responses to haptens that persisted for 28 days and was elicited only by haptens to which mice were previously sensitized [7]. No contact hypersensitivity was induced in another type of mouse lacking NK cells (and T and B cells), suggesting that NK cells were mediating a true adaptive secondary response [7]. Further, adoptive transfer studies indicated NK cells were the specific leukocyte involved. However, a specific receptor or mechanism imparting this memory was not found [7]. Another investigation of the involvement of NK cells in epidermal responses suggested that NK cells do not mediate specific memory in a murine skin transplant model, but will mediate acute skin allograft rejection after IL-15 stimulation in the absence of any adaptive immune cells [26].

Sun and Lanier utilized B6 mice and the mouse cytomegalovirus to investigate the role of NK cells in a viral infection [8]. Following initial infection, NK (Ly49H receptor+) cells proliferated 100x in the spleen and 1000x in the liver. After a contraction phase, these NK cells resided in various tissues for several months. Following secondary exposure, or viral reactivation, memory NK cells were found to rapidly degranulate and produce cytokines, resulting in protection. Adoptive transfer of these NK cells also conveyed protective immunity. For the first time, immune responses of NK cells were found to undergo all four phases: expansion, contraction, memory maintenance and recall response, previously attributed only to cells of the adaptive immune system.

Unlike T and B cells that express one antigen-specific type of receptor after encountering a pathogen, NK cells have been found to express an array of receptors with distinct specificity [8], [27]. Natural Killer cells may preserve a more general adaptive protection similar to what is observed in memory T cells, where interleukin-12 produced by dendritic cells triggers interferon-gamma production in the absence of cognate antigen [28].

Receptors on mammalian NK cells that could provide memory function and cross-reactivity have been discussed [25], [28], [29]. Human NK cells can be activated by direct contact with *Mycobacterium* via the NKp44 natural cytotoxicity receptor [30]. This activation occurs in the absence of monocytes/macrophages and IL-12. In bony fish the NK cell receptor functional orthologs are novel immune-type receptors (NITRs) [31], [32]. Like NK cell receptors in mammals, NITRs can function to either inhibit or activate NK cell cytotoxicity and/or cytokine release [32]. A group of secreted NITRs have been suggested to dimerize with membrane-bound NITRs or other membrane-bound molecules involved in immune recognition, or they may bind to foreign body surfaces [33]. The structure of an activating NITR on a cytotoxic NK-like cell line has been characterized as resembling antigen binding receptors that demonstrate specific recognition, and these receptors might undergo lineage-restricted somatic variation conveying specific protection upon re-encounter with the same pathogen [31]. Based on our studies that have demonstrated specific secondary immune responses of T and B cell deficient rag1^−/−^ mutant zebrafish, and evidence that fish NK cells demonstrate the capacity of specific recognition of diverse molecules [31], we believe that a population of zebrafish NK cells mediate memory, and that zebrafish NK cells have a mechanism for enhanced discrimination of a bacterial target following primary exposure.

In addition, other receptors have been shown to be important in recognizing intracellular pathogens. Two are of particular interest, the tripartite motif (TRIM) proteins and NOD-like receptor (NLR) molecules. Both have been shown to be present and very diverse in zebrafish. The zebrafish genome encodes 240 TRIMs [34]. Many of them have the B30.2 domain and an important ligand binding domain [35]. This region displays a high level of positive selection for diversification in zebrafish [35]. The zebrafish genome also encodes 5 NLR A family members, 6 NLR B family members and several hundred NLR C family members [36]. Like the diverse TRIMs, the NLR Cs contain the B30.2 domain with high diversity [36]. Involvement of cytosolic receptors in the host response to *E. ictaluri* is suggested by the demonstration of up regulation of the NLR designated NOT1 in infected channel catfish [37]. Because these receptors are cytosolic, any protection imparted would likely be phagocyte mediated.

### Conclusion

We used rag1^−/−^ mutant zebrafish to examine immunological memory of the innate immune system to a bacterial pathogen. Memory is a term that has been only used in acquired immunity therefore we referred to innate immune system memory as adaptive protection throughout this text. Heterologous bacterial challenges demonstrated that the immune system of rag1^−/−^ mutant zebrafish exhibits adaptive characteristics and specificity. Adoptive cell transfers demonstrated that kidney interstitial cells mediated the specific adaptive protection. Our research demonstrates that innate immune cells are capable of mediating specific adaptive protection and that vaccination in rag1^−/−^ mutant zebrafish results in significantly increased survival if the fish are re-exposed to the same pathogen.

Even if it is ultimately revealed that adaptive protection is more important in fish than mammals, understanding this immune response in fish will help to understand its phylogenetic development. The broader spectrum provided by memory of innate immunity would influence an individual’s ability to respond to classes of pathogens and direct the type of acquired response induced. Innate memory could be partially responsible for variation and alterations of immune function in immune-associated diseases and could be directed to help with deficiencies in acquired immune components.

## Supporting Information

Figure S1 **Lethal Dose (LD) trials for Edwardsiella ictaluri, Edwardsiella tarda and Yersinia ruckeri in rag1^−/−^ mutant and wild-type zebrafish.** Four replicate tanks per treatment with 15 fish per replicate were injected with indicated dosages of bacteria and 15 control fish per strain were sham injected with PBS. Mortalities were recorded for 18 days post injection (DPI). No mortalities were observed in the control fish.
(TIF)

Figure S2 **The experimental design of Trial 5, Adoptive cell transfers in rag1^−/−^ mutant zebrafish.**
(TIF)
