# Supplementary figures and images for: Rag1−/− Mutant Zebrafish Demonstrate Specific Protection following Bacterial Re-Exposure
Source: PLoS One. 2012 Sep 6;7(9):e44451. doi: 10.1371/journal.pone.0044451 (PMC3435260; doi:10.1371/journal.pone.0044451)

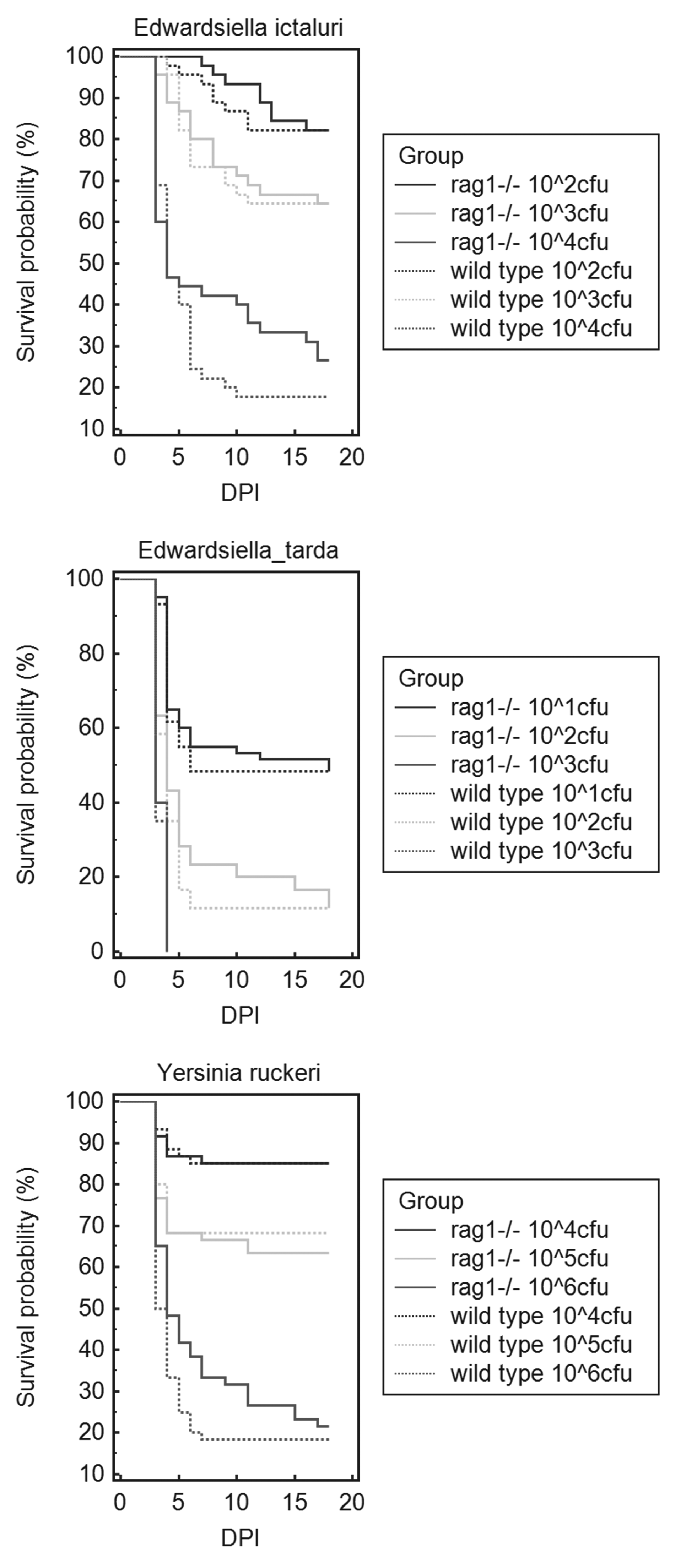

Supplement: Figure S1 — Lethal Dose (LD) trials for Edwardsiella ictaluri, Edwardsiella tarda and Yersinia ruckeri in rag1−/− mutant and wild-type zebrafish. Four replicate tanks per treatment with 15 fish per replicate were injected with indicated dosages of bacteria and 15 control fish per strain were sham injected with PBS. Mortalities were recorded for 18 days post injection (DPI). No mortalities were observed in the control fish. (TIF) [file pone.0044451.s001.tif]
